# Comparison of human and bacterial monofunctional catalase structures obtained by electron cryomicroscopy

**DOI:** 10.1107/S2052252526008845

**Published:** 2026-11-03

**Authors:** Daria Slowik, Jade Li, Hugh Wilson, Alexander Shtyrov, Shaoxia Chen, Greg McMullan, Christopher J. Russo, Garib Murshudov, Richard Henderson

**Affiliations:** a MRC Laboratory of Molecular Biology, Francis Crick Avenue, CambridgeCB2 0QH, United Kingdom; Max Planck Institute of Molecular Physiology, Germany

**Keywords:** haem, NADPH, radiation damage, peroxidase, cryoEM, X-ray structure

## Abstract

We present high-resolution (1.7–1.9 Å) structures of three monofunctional catalase enzymes from the clade 3 grouping, including two bacterial and one human erythrocyte catalase, all determined by single-particle electron cryo­microscopy (cryoEM). The haem environment of all three structures is conserved; the fully occupied NADPH binding site shows slight differences, yet the ordered water structure varies and there are interesting differences in radiation damage to aspartic acid and glutamic acid side chains. We also compare these cryoEM structures with previous X-ray crystal structures. The C-terminus of the cryoEM structure of *M. luteus* catalase in solution has 16 disordered residues which are well ordered in the X-ray crystal structure. The ordered C-terminus in the X-ray structure may be caused by crystal contacts or by the 30 times higher ionic strength of 1.4 *M* ammonium sulfate used for crystallization, compared with 0.15 *M* phosphate-buffered saline used in cryoEM. Conversely, the bound NADPH is better ordered and strongly bound in the cryoEM map. It is displaced, probably by the sulfate ions, in the crystal. These structures demonstrate the strength of cryoEM in which the maps show the structure directly so that potential bias in the maps used for model refinement is absent.

## Abstract 2

Three high-resolution catalase structures have been determined by cryoEM, two bacterial and one human. Comparison of the haem and NADPH cofactor binding, water molecules and channel structure between these and previous X-ray crystal structures provides insight into the different features of the structures and the strengths of the methodologies.

## 1. Introduction

Catalases are redox enzymes that are responsible for the dismutation of hydrogen peroxide into water and molecular oxygen. Dismutation is an activity in which a single compound is simultaneously oxidized and reduced to form two different compounds, with higher and lower oxidation states than the starting material. Catalase converts two molecules of hydrogen peroxide (H_2_O_2_) into two molecules of water plus one molecule of oxygen. Formally it is therefore a hydrogen-peroxide:hydrogen-peroxide oxidoreductase (EC 1.11.1.6). Its two-step mechanism is as follows, where Fe^III^ represents the haem iron in catalase ground state and O=Fe^IV·+^ represents the oxyferryl catalase (radical cation ·+) intermediate called compound I.
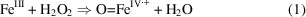

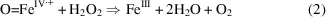


There are many primary publications and reviews that provide an overview of our current understanding of catalase structure and function (Díaz *et al.*, 2012 ▸; Kirkman & Gaetani, 2007 ▸; Reedy & Gibney, 2004 ▸). The enzyme was first identified and named catalase by Loew (1900 ▸) and was one of the first proteins to be crystallized by Sumner & Dounce (1937 ▸). Many thousands of X-ray crystal structures of diverse species of catalase have been determined at increasing resolutions (Murshudov *et al.*, 1992 ▸; Murshudov *et al.*, 2002 ▸; Putnam *et al.*, 2000 ▸) after the first three-dimensional structure determinations at 3.5 Å and 2.5 Å resolution by Vainshtein *et al.* (1981 ▸) and Murthy *et al.* (1981 ▸), respectively. Klotz *et al.* (1997 ▸) constructed a phylogenetic tree based on an analysis of 70 catalase sequences and identified three clades. Díaz *et al.* (2012 ▸) reviewed 30 years of catalase structural biology drawing attention to the differences between the monofunctional catalases with their highly conserved three-dimensional fold (the catalase fold) and dual function catalase-peroxidases which have a completely different three-dimensional fold (Gómez *et al.*, 2019 ▸) yet are still haem-based enzymes that efficiently catalyse dismutation of hydrogen peroxide as well as the peroxidase reaction of H_2_O_2_ with various organic electron donors. In this work we determined the cryoEM structures of three catalases in clade 3. Clade 3 is the group of monofunctional catalases that are homo-tetramers with *D*2 symmetry, having small polypeptide chains of 450–525 residues, haem b and NADPH cofactors, a tyrosine proximal haem ligand and conserved distal histidine and asparagine residues in the active site.

There are eight unique monofunctional catalase structures from clade 3 (Klotz *et al.*, 1997 ▸) with resolutions better than 2.0 Å (Table 1 ▸) deposited in the Protein Data Bank (PDB), all determined by X-ray crystallography. In contrast, there are only two structures of human erythrocyte catalase deposited in the PDB obtained using cryoEM, both at lower resolutions. A 2.2 Å resolution structure was determined as part of a project to investigate the behaviour of human erythrocyte catalase at the air–water interface (Chen *et al.*, 2022 ▸) and the other was one of 11 structures determined to demonstrate the desirability of cryoEM at 100 keV (Naydenova *et al.*, 2019 ▸; McMullan *et al.*, 2023 ▸) but at lower (3.4 Å) resolution.

The original purpose of the work we present here was to obtain accurate high-resolution cryoEM 3D maps so that the electron scattering form factors for the atom types H, C, N, O, P, S and Fe in different bonding arrangements could be experimentally determined (Shtyrov *et al.*, 2026 ▸). Clade 3 catalases were selected because they contain both a haem group with its iron atom and three phosphate linkages in NADPH. Until now, the traditional form factors for electron scattering are those originally tabulated for independent (*i.e.* non-bonded) atoms in the *International Tables for Crystallography* (Prince, 2006 ▸). These are currently used during refinement of atomic models to fit the potential maps that are determined experimentally by cryoEM. CryoEM has resulted in over 30000 PDB depositions, and all have had their coordinates refined using traditional form factors for non-bonded atoms, as tabulated. The maps described here have been used to obtain experimental parameters for form factors, which should be more realistic, thus improving the map–model Fourier shell correlation (FSC), lowering the *R* factors and leading to more accurate atomic coordinates (Shtyrov *et al.*, 2026 ▸).

One of the problems encountered by cryoEM in the earlier years arose because of beam-induced motion or blurring in the first few frames of the CMOS-detector-based movies (Henderson, 2015 ▸). This caused the contributions to the cryoEM maps from the early least-damaged frames of the movies, consisting of the first few e^−^ Å^−2^ of the exposure, to be largely wiped out in the final map, making any attempt at form factor determination pointless. It has been the introduction of small-hole (<300 nm diameter) gold grids (Naydenova *et al.*, 2020 ▸) to prevent or greatly reduce this beam-induced motion in the first few movie frames that has enabled this project to be contemplated. These gold grids, particularly with the small holes we have used, are the key that underpins the work described in this paper that could not have been carried out before this recent innovation.

After examining the 3D cryoEM maps of the three catalase structures, it became clear that they had many interesting features. The high resolution allowed many water molecules and alternative conformations to be unambiguously described, and the extent of radiation damage in cryoEM structures could be observed. Although there are still potential errors and overfitting problems during atomic coordinate refinement and interpretation due to wrong modelling or suboptimal dictionaries, the fact that cryoEM maps are entirely experimentally determined means that the impact of model bias or dictionary perturbations is lower, especially for ligands or cofactors such as haem or NADPH. The purpose of this paper is to describe and compare these three new cryoEM structures with one another and with two earlier X-ray crystallographic structures for the human erythrocyte (Putnam *et al.*, 2000 ▸) and *Micrococcus luteus* (Murshudov *et al.*, 2002 ▸) catalases, which were determined previously at resolutions of 1.5 Å and 0.88 Å, respectively, and which represent the highest resolutions available to date for these two catalase structures. The three catalase enzymes studied here are *Micrococcus luteus* catalase (cat_L), *Rhizobium radiobacter* catalase (cat_T), and human erythrocyte catalase (cat_H).

## 2. Materials and methods

### 2.1. Cloning, expression and purification of *Micrococcus luteus* catalase

An initial attempt was made to use *Micrococcus luteus* catalase purchased from Sigma-Aldrich (100 ml, Cat. No. 60634). A test cryoEM session gave good specimens and good micrographs, but subsequent 3D classification showed that only 25% of the material was the desired catalase structure. A major impurity gave a completely different sub-3 Å structure for a similar-sized molecule that also had *D*2 symmetry; this was identified as an aldehyde de­hydrogenase using *ModelAngelo* (Jamali *et al.*, 2024 ▸). The full DNA coding sequence of the *catA* gene (Murshudov *et al.*, 2002 ▸) was synthesized and cloned into the pUCIDT-Amp GoldenGate vector (Integrated DNA Technologies, IDT). The construct included an N-terminal 8× histidine tag, separated from the *catA* sequence by a flexible serine–glycine linker. The sequence was subsequently subcloned into the pCOLD III vector (TaKaRa) at the NdeI/XbaI sites of the multiple cloning site (MCS). Correct insertion and sequence integrity were confirmed by Sanger sequencing (Eurofins).

Protein expression was carried out in *E. coli* BL21(DE3)pLysS cells (Novagen). Cultures were induced with 0.5 m*M* IPTG (isopropyl β-d-1-thiogalactopyranoside, Cambridge Bioscience) at an optical density at 600 nm (OD_600_) of 0.5 and incubated for 10 h at 18 °C with shaking at 200 rpm. Cells were harvested by centrifugation at 10000*g* for 20 min at 4 °C, resuspended in phosphate-buffered saline (PBS), pH 7.4, supplemented with cOmplete EDTA-free protease inhibitor tablets (Roche), and lysed by sonication on ice. All subsequent steps were performed at 4 °C.

The soluble protein fraction was obtained by sequential ultracentrifugation at 200000*g* for 45 min and 400000*g* for 30 min, using Ti45 and Ti70 rotors (Beckman Coulter), respectively. Catalase was further purified using an Ni-NTA Superflow cartridge (Qiagen) in PBS (pH 7.4) supplemented with 0.5 *M* NaCl and 40 m*M* imidazole. The protein was eluted with 250 m*M* imidazole in PBS. The eluted sample was dialyzed against buffer A (20 m*M* Tris–HCl, pH 7.0) containing 1 m*M* EDTA and further purified by anion exchange chromatography on a HiTrap DEAE FF column (Cytiva) using a linear gradient of 0–1 *M* NaCl in buffer A. Eluted fractions were supplemented with 40 m*M* imidazole and subjected to a second purification step on a HisTrap HP column (Cytiva). Catalase was eluted using a linear gradient of 40–400 m*M* imidazole in buffer A. Prior to elution, all columns were thoroughly washed with the appropriate binding buffer to remove non-specifically bound proteins. The final yield was 2.04 mg from 12 litres of culture.

Fractions containing catalase were pooled, concentrated and subjected to size-exclusion chromatography on a Superdex 200 column (Cytiva) equilibrated in PBS. Final protein concentration to ∼20 mg ml^−1^ was achieved using an Amicon Ultra-100K centrifugal filter unit (Millipore), followed by addition of NADPH to 0.2 m*M* and CHAPSO to 5 m*M*.

### 2.2. Cloning, expression and purification of *Rhizobium radiobacter* catalase

The catalase from *Rhizobium radiobacter* was chosen because it was shown to be highly thermostable (Hossain *et al.*, 2008 ▸), which may minimize denaturation at the air–water interface. The full DNA coding sequence of the *katH* gene (Hossain *et al.*, 2008 ▸) was synthesized (Integrated DNA Technologies, IDT), and subsequently cloned, expressed and purified using the same procedure as described for *Micrococcus luteus* catalase. The final yield was 0.88 mg from 12 litres of culture.

### 2.3. Human erythrocyte catalase

Human erythrocyte catalase was obtained from Sigma (C3556) at a concentration of 2 mg ml^−1^. The protein was concentrated to 25 mg ml^−1^ and the buffer changed to PBS (pH 7.4) using an Amicon Ultracel 100k centrifugal filter spin column. NADPH (β-nicotinamide adenine dinucleotide 2′-phosphate reduced tetrasodium salt hydrate, Sigma-Aldrich, N7505) was added at 0.2 m*M* prior to the final concentration step. The detergent 3-(3-cholamido­propyl-di­methyl­ammonio)-2-hy­droxy-1-propane­sulfonate (CHAPSO) was obtained from Sigma (C3649) and added to the sample to a final concentration of 5 m*M*. The sample applied to grids thus consisted of 25 mg ml^−1^ human erythrocyte catalase in PBS, pH 7.4 with 0.2 m*M* NADPH and 5 m*M* CHAPSO.

### 2.4. Single-particle cryoEM specimen preparation

Catalase specimens for single-particle cryoEM were prepared on HexAuFoil supports (Naydenova *et al.*, 2020 ▸; Naydenova & Russo, 2022 ▸) by plunge freezing (Passmore & Russo, 2016 ▸) with a Talmon-type manual plunger (Bellare *et al.*, 1988 ▸). HexAuFoil grids have 0.3 µm holes separated by 0.3 µm gaps on a hexagonal lattice. Immediately prior to plunge freezing, the grids were treated with a 9:1 Ar:O_2_ plasma to render the surface hydro­philic (60–90 s, 30 standard cubic centimetres per minute, 40 W forward power, Fischione Instruments, 1070 Nanoclean). For each specimen, a 3 µl droplet of protein solution with 0.2 m*M* NADPH and 5 m*M* CHAPSO detergent was applied to the foil side of a plasma-treated grid and blotted with filter paper (Whatman No. 1) from the foil side for 15–30 s. The grid was then immediately plunged into liquid ethane held at 93 K in a temperature-controlled cryostat (Russo *et al.*, 2016 ▸). This was carried out in a 4 °C cold room, with the grid environment equilibrated to greater than 90% relative humidity to reduce evaporation from the thin film of catalase solution in the short gap between blotting and freezing.

### 2.5. Data collection and processing

Movies were acquired on a Titan Krios microscope operating at 300 kV using a Falcon 4i detector in counting mode, under the control of *EPU* software version 3.7.0 (Thermo Fisher Scientific). The nominal magnification was 155,000×, with a pixel size of 0.512 Å calibrated using the Au reflections present in a selection of acquired images due to the presence of support foil within the field of view (Dickerson *et al.*, 2024 ▸). A 100 µm objective aperture was used during data collection. Movies were acquired with 3.0 s exposure time, and the flux was set to 18 e^−^ Å^−2^ s^−1^ giving a total fluence of 54 e^−^ Å^−2^. Raw frames in EER format were combined in groups of 32 to give 28–30-frame movies with approximately 1.8 e^−^ Å^−2^ per movie frame.

The three datasets were acquired using the Krios aberration-free image shift (AFIS) option, with successive refinement of the maximum image shift radius to optimize the efficiency of data collection, to limit the beam tilt variation and to minimize the number of optical groups required. The maximum AFIS image shift for cat_T was 12 µm, which required 12 optical groups; for cat_H and cat_L, it was 1.5 and 3.5 µm, respectively, requiring only one optical group. It is also notable in Table 2 ▸ that the beam tilt correction for cat_T, at 0.5 mrad, was greater than for cat_H or cat_L. The supplementary figures show the orientation distribution (Fig. S1), the FSC plots (Fig. S2) and the increasing *B* factor with exposure (Fig. S3). It is notable that the first frame in each case showed the lowest *B* factor, as expected from the use of the HexAuFoil gold grids (Naydenova *et al.*, 2020 ▸). Fig. S4 shows the particle distribution in a typical micrograph from each of the three datasets.

The electrostatic potential maps were reconstructed from the acquired movies using *RELION-4* (Zivanov *et al.*, 2022 ▸). First the movies were motion corrected with *MotionCor2* (Zheng *et al.*, 2017 ▸), and the contrast transfer functions (CTFs) were fitted using *CTFFIND4* (Rohou & Grigorieff, 2015 ▸). Particles were manually picked and used to train a *Topaz* (Bepler *et al.*, 2019 ▸) model which was then used for auto­picking. *RELION-4* was used for 2D and 3D classification, 3D refinement with *D*2 symmetry, CTF refinement, particle polishing, and post-processing. Statistics are given in Table 2 ▸.

### 2.6. Model building and refinement

Models were built following rigid-body docking of PDB depositions (1GWE for cat_T and cat_L, and 7P8W for cat_H) into the sharpened maps from *RELION*. The 1GWE model was expanded to the tetramer and docked into the *RELION* maps for *R. radiobacter* and *M. luteus* catalases. For cat_T and cat_L, the bound ions, water molecules and protein side chain alternate conformations in 1GWE were deleted before model building into the cryoEM map. For cat_H, the 7P8W model was docked into the *RELION* map for the human catalase. For cat_H, the protein alternate conformations were deleted from 7P8W before model rebuilding, but of the bound water molecules roughly two-thirds found in agreement with the new cryoEM map were retained. The models were then manually adjusted, with sequence correction for *R. radiobacter*. Docking and rebuilding were performed in *Coot* (Emsley *et al.*, 2010 ▸). Around 400 water molecules were added to each of the three structures, slightly more for cat_T with slightly higher resolution (Table 2 ▸). The models were refined against the unsharpened halfmaps using *Servalcat* (Yamashita *et al.*, 2021 ▸). The main figures and Figs. S5–S9 were made using *Coot* screenshots.

## 3. Results and discussion

### 3.1. The 3D maps

The plots of *B* factors versus movie frame (Fig. S3) for each of the structures show the benefit of the small-hole HexAuFoil gold grids (Naydenova *et al.*, 2020 ▸) as indicated by the fact that the lowest *B* factors are in the first frames, and that the FSC plots extend to resolutions of 1.87, 1.92 and 1.73 Å for cat_H, cat_L and cat_T maps, respectively, using the FSC = 0.143 resolution criterion (Rosenthal & Henderson, 2003 ▸). The plots of the scale factors (intercepts at zero spatial frequency) for the different movie frames (Fig. S3) decrease from 1.0 to around 0.7 at the end of the exposures; this is generally thought to be due to ∼30% mass loss due to radiation damage. Fig. 1 ▸(*a*) shows the density for the haem and Fig. 1 ▸(*b*) for NADPH in cat_T. Figs. 1 ▸(*c*) and 1 ▸(*d*) show two typical tryptophan residues in cat_T. Table 2 ▸ gives the statistics of data collection and processing. The three catalase maps in this work can be compared with the statistics of all EMDB depositions using a plot of number of asymmetric units against resolution (Patwardhan *et al.*, 2026 ▸). They lie at the upper edge of resolutions attained for the number of asymmetric units used, so the overall quality of these three maps is good. The efficiency of orientation distribution *E*_od_ (Naydenova & Russo, 2017 ▸) is 0.72–0.75 (Table 2 ▸) thanks to the use of CHAPSO to create detergent monolayers to prevent preferential orientation by adsorption to the air–water interface during blotting. The *Q*-scores (Pintilie *et al.*, 2020 ▸) for the map–model fit were all above 0.8 (Table 2 ▸).

At the N-terminus, the map densities begin at S4(H), T7(L), G10(T) in the three structures. In the corresponding X-ray structures of cat_H and cat_L, the densities start at R5(H) and T6(L), basically the same within one residue. The first residues that are structurally homologous between human and bacterial enzymes are S13(T,L) with L27(H), followed by the identical threonines T14(T,L) and T28(H) in all three structures. A few residues further, at prolines P20(T,L) and P34(H), the structures approach within 1 Å, thereafter becoming ever more similar. *Cis*-proline P60 in cat_T and cat_L overlaps precisely with V74 in cat_H; both are in van der Waals contact (3.5–3.7 Å) with the haem. Proline P108(T,L) in cat_T and cat_L is replaced by alanine A123(H) in cat_H, while W111(T,L) is replaced by V126(H). In cat_T and cat_L, P108(T,L) is stacked with W111(T,L) across a four-residues turn; in cat_H, a water molecule occupies the space between the smaller side chains (Fig. S5). *Cis*-prolines P389(L) and P406(H) are replaced by T389(T) in cat_T (Fig. S6), with 1.3 Å backbone distance between the two *cis*-proline positions; this is a region adjacent to a surface loop deletion in cat_H where the 19/18 residue sequence S395-F413(T) and P412-V429(H) is divergent between human and bacterial catalases. This is followed by another surface loop with a deletion of three residues in cat_H leading to different conformations for the 8/5 residues A417-D424 (T,L) and N433-D437(H). Finally, there is another loop-out with two adjacent glycines in cat_T (G456-G457) and only one in cat_L (Fig. S7), with an approximate return to being in register but with weaker similarity between bacterial and human sequences right through to the C-terminus.

At the C-termini, the densities in all three maps fade out within the same three residues in three dimensions. Q489(T), H487(L) and E503(H) show the last visible density. The X-ray structure 1DGF for cat_H has no density beyond N501(H), so there is slightly better resolution at the C-terminus in the cat_H cryoEM map. However, the X-ray structure 1GWE for cat_L shows an additional 16 residues compared with the cat_L cryoEM map, which will be discussed later.

### 3.2. Haem environment

The density for the haem in the centre of each subunit in all three catalase maps shows an almost perfect representation of haem b density with no evidence of radiation damage [Figs. 1 ▸(*a*) and 2 ▸]. The two bacterial haems are similar (0.13 Å maximum distance) but the human catalase haem and protein together have a slight tilt (about 5°) relative to the 222 symmetry axes. P60(L,T) pushes against the haem (Fig. 2 ▸), whereas the homologous V74(H) is 1.5 Å more distant.

The proximal tyrosine ligands Y343(L,T) and Y358(H) are also clear with Fe—O bond lengths of 1.88, 1.98 and 1.92 Å (H,L,T) and atomic positions within 0.39 Å. The distal catalytic sites all have the essential histidine H61(L,T), H75(H) and asparagine N133(L,T), N148(H).

There are two water molecules in the active-site cavity in identical positions in all three catalase structures. Water W971(H)/W910(L)/W860(T) is 2.5–2.6 Å from the haem iron. The second water W889(H)/W903(L)/W825(T) is hydrogen-bonded (at 3.1 Å distance) to N133(L,T)/N148(H) and (at 2.8 Å distance) to NE2 of H61(L,T)/H75(H) and (at 2.6 Å distance) to the haem water. The density for the haem water is lower (at 1.0, 1.4 and 1.3 σ for H, L and T, respectively) than for the more distant water (2.7, 2.8 and 2.9 σ), suggesting 40–50% occupancy. Both haem cavity waters are found in similar positions in the *M. luteus* 1GWE X-ray structure, with similar full occupancy for the water that is coordinated to the essential His/Asn and similar partial occupancy for the haem water. Only the full occupancy water is included in the human 1DGF X-ray coordinates together with a statement (Putnam *et al.*, 2000 ▸) that water is discriminated against at the sixth haem iron coordination site. There is also density at ∼2.4 Å from the haem iron in the 1DGF map at ∼40% occupancy. In the three cryoEM maps, the partially occupied haem water is clear.

In unliganded catalase from *C. glutamicum* 4B7G, on the distal side of the haem the same two water molecules are observed between the essential histidine and asparagine and at the haem iron (Candelaresi *et al.*, 2013 ▸). They also observed irradiation-induced photoreduction at a dose of 0.5 MGy in the nitric oxide liganded structure and adopted measures to mitigate it by collecting a full dataset at much lower dose. Photoreduction, or its equivalent for electron radiation, is a problem likely to be more significant because of the typically greater dose imparted to the specimen.

The two haem propionate groups each have arginine counterions; R350(T,L), R365(H) is the counterion to CGA carboxyl and R97(T,L), R112(H) is the counterion to CGD carboxyl. The position of the haem vinyl group (CBC), the adjacent serine residue (S217 in cat_H), and the channel leading to the NADPH nicotinamide are similar in all three structures, so the compound II reduction mechanism proposed by Sicking *et al.* (2008 ▸) is consistent with all three structures.

### 3.3. NADPH binding site

The NADPH conformation and surrounding interactions seen in Fig. 3 ▸ are similar in the three structures but have slight differences. For example, arginine R188(L,T) and R203(H) form conserved salt bridges with the NADPH (P2B) phosphate and there is a conserved water molecule with the guanidinium stacked on the adenine ring. A second arginine R295(L) in *M. luteus* catalase is also an NADPH phosphate (P2B) counterion with N–O distances of 2.71 and 2.85 Å, yet a leucine is the homologous residue L295(T) and L310(H) in cat_T and cat_H with H222(T) and K237(H) providing the equivalent charge compensation. Surprisingly, cat_T is more similar to cat_H than to cat_L, whereas cat_L and cat_T have closer percentage sequence identity overall (Table S1). There is also a big difference between Y183(T,L) and F198(H) with a 120° β−γ bond rotation to put the side chain in a different orientation, pushing the NADPH by ∼1.8 Å into a new position. The side chain of F198(H) is in contact (3.4 Å) with the NADPH adenine in cat_H whereas Y183(T,L), although still making contact with the adenine (3.6 Å), is oriented towards the start of the pathway between NADPH and the haem.

Sicking *et al.* (2008 ▸) and Korth *et al.* (2012 ▸) discussed the controversial role of NADPH in clade 3 catalase activity. Side reactions at the midpoint of the oxidoreduction reaction, after one molecule of hydrogen peroxide has generated the O=Fe^4+·^ oxyferryl compound I intermediate, produce the inactive compound II via one-electron reduction especially at low H_2_O_2_ concentrations. NADPH, 13–19 Å distant from the haem, then either prevents or rescues catalase compound II to restore the fully active ferricatalase resting state. Korth *et al.* (2012 ▸) showed how the bound NADPH after reaction to produce NADP^+^ is then re-reduced by external (unbound) NADPH rather than by NADPH–NADP^+^ exchange. The subtle variations in how NADPH is bound in the different catalase structures do not seem to affect its important role as a protective cofactor.

### 3.4. Locations where cat_T is closer to cat_H than to the other bacterial cat_L

The pairwise sequence identity for cat_T, cat_H and cat_L is shown in Table S1. One hundred and ninety-one residues are identical in all three catalases (38%). Although the two bacterial catalases cat_T and cat_L are closest to one another with 68% identity, there are 54 sequence positions (11%) where one of the bacterial enzymes is identical to human, yet different from the other bacterial sequence: cat_T and cat_H are closest with 34 identical residues in locations where cat_L differs, but there are only 20 residues where cat_L and cat_H are identical and cat_T different. Given these genetic differences, we looked in detail at the positions where cat_L is unique.

The NADPH binding site, described above, is one location where cat_T is closer to human cat_H than its more closely related cat_L. Proline P32(T) and P46(H) is serine in cat_L with almost identical backbone conformation. Proline P147(T) and P162(H) is a threonine in cat_L with close superposition. Proline P289(T) and P304(H) is replaced by serine S289(L), and is near NADPH but with side chains directed away from it. Proline P385(T) and P402(H) are replaced by serine in cat_L with backbones within 1 Å.

N26(T)/N40(H) forms a hydrogen bond with K334(T)/K349(H)-NH1, whereas Q26(L) forms a hydrogen bond with R334(L)-NH1. In another correlated sequence change, A48(T)/A62(H) is replaced by Q48(L) and the space occupied by Q48(L) is filled by R348(T)/R368(H).

### 3.5. Two channels leading to the surface

One longer and wider channel of length roughly 25 Å from the haem iron to bulk solvent (shown in Fig. 4 ▸) is the path thought to be taken by H_2_O_2_. Similar diagrams of this channel from the two X-ray crystal structures are shown for cat_H in Fig. 3(*a*) of Putnam *et al.* (2000 ▸) and for cat_L in Fig. 7 of Murshudov *et al.* (2002 ▸). We observe more water molecules in cat_H and fewer in cat_L than in the X-ray structures, possibly due to a more conservative approach in the cat_H X-ray structure determination and a higher resolution in the cat_L X-ray structure. At the surface end of the haem channel, in the space occupied by W111(T,L), there is a cavity in human catalase near V126(H), filled with low-occupancy water molecules. Also, the ordered C-terminal residues in the cat_L X-ray structure approach the entrance to the channel in the same subunit, replacing some of the water molecules.

A second pathway or channel for electrons must connect the haem to NADPH with a 13.1 Å distance between the nicotinamide hydrogen and the haem vinyl. This distance is shorter than the length of the haem channel and it is known that electrons can efficiently tunnel between redox centres (Kaila *et al.*, 2010 ▸). While the potential route for this electron trajectory from the NADPH to the haem is not clear, it may involve Y200(T,L)/Y215(H) (Olson & Bruice, 1995 ▸) and/or S202(T,L) (Sicking *et al.*, 2008 ▸).

Two other papers describing X-ray structures also discuss the nature of the channels. Candelaresi *et al.* (2013 ▸) mention four ordered water molecules in the 25 Å haem solvent channel that leads to the surface of the protein, fewer than observed in the three cryoEM maps. They also observe a water molecule as the sixth ligand to the haem (iron–water distance of 2.3 Å) confirming that this is likely to be the situation in all resting catalases. Sevinc *et al.* (1999 ▸) consider the nature of the NADPH lateral channel in *E. coli* HPII, which is a clade 2 catalase with a longer C-terminal extension. It does not bind NADPH yet its R260A mutation (equivalent to R188 in cat_L, cat_T, which forms a salt bridge with NADPH P2B) resulted in a threefold increase in catalase turnover rate, suggesting that H_2_O_2_ may be able to reach the haem catalytic centre by more than one route.

### 3.6. Alternate conformations

Seven protein residues in cat_H, 14 in cat_L and 19 in cat_T are present in alternate conformations as revealed by bifurcated densities. Since the cryoEM maps are entirely experimental, they provide direct evidence that alternate conformations may be quite common even though they may not be resolved at a given resolution. Two examples of residues with alternate conformations are shown in Fig. S8.

V41(T) has a clear dual conformation, with only a single conformation being present in homologous T41(L) and V55(H). The backbone at G106(T,L), G121(H) has alternate conformations with peptide flipping between the two states in all three structures and breaking the symmetry. Leucine L130(T,L) has a dual conformation, whereas the homologous L145(H) has a single state. The side chains of several lysine, arginine, aspartate, glutamate or glutamine side chains are either bifurcated or differ between catalases, including K154(T,L), K261(L), D404(T), D410(T,L), E448(T), Q462(T,L)/Q475(H), E484(H), R465(T), E476(T). At an inter-subunit contact, tyrosine Y378(T,L) in subunit A has a dual conformation and makes contact with Y378(T,L) in subunit B, so the two residues in adjacent subunits flip-flop together and break the strict *D*2 symmetry, with an occupancy of 0.5 in both locations (Fig. S8). Other examples of alternate conformations include L119(T,L)/V134(H), L301(L)/L316(H), F117(T,L)/F132(H) and F221(T,L)/Y236(H).

### 3.7. Salt bridges

There are about 40 salt bridges between positively and negatively charged side chains in each structure, so these are dominant features. Partly, this high incidence is due to the haem and NADPH cofactors, each of which has three intrinsic charge centres. The haem is buried and the NADPH is on the surface, but each uses charge compensation to stabilize well defined conformations. These six cofactor charge centres account for six of the salt bridges, but there are many others.

The side chains of R192(T) and R192(L) differ by rotation about their γδ bond, with both having well resolved density. They form different salt bridges with E228(T) and E196(L), respectively, with good density for both. The corresponding human residue D207(H) forms a salt bridge to K243(H). This is an example of a correlated sequence change with inverted charges (Fig. S9).

### 3.8. Radiation-damaged aspartate and glutamate side chains

The exposure in the first and subsequent frames of the images used for data collection in this work was about 1.8 e^−^ Å^−2^, which corresponds to a dose in the protein of 4 to 5 MGy. Candelaresi *et al.* (2013 ▸) observed significant photoreduction at the haem in their X-ray crystallographic work on the binding of nitric oxide to *Corynebacterium glutamicum* catalase at a similar dose. Therefore with cryoEM as currently practised and as carried out in this work, we are well into the dose range where radiation damage to glutamate, and to a lesser extent aspartate side chains, and electron reduction of haem is significant.

There are around 20 mainly glutamate and occasionally aspartate side chain densities that are weak or absent, suggesting the presence of either disorder or radiation damage. The high-resolution X-ray structures of cat_L and cat_H helped to identify which residues were more likely to be radiation damaged than disordered. Examination of the 3D structures calculated only from the first movie frame can distinguish between disorder and radiation damage. Structural disorder affects all frames, whereas side chains prone to radiation damage show up more clearly in the first frame than in subsequent frames. A difference map between the first and second frames of the movie shows radiation damage density loss on many glutamic acid carboxyl­ate groups (*e.g.* Fig. 5 ▸), plus a few on aspartic acid and me­thio­nine side chains. The density loss between frames 1 and 2 (cumulative fluence 0 to 1.8 e^−^ Å^−2^ and 1.8 to 3.6 e^−^ Å^−2^) on glutamate carboxyl­ate groups amounts to 25–75% of the initial density with the percentage change on aspartic and me­thio­nine side chains being 3–4 times less than for glutamic acid. A more detailed analysis of radiation damage in these three catalase structures will be the topic of a separate paper focusing entirely on radiation damage. It would also be useful in future to carry out a much larger (and longer) data collection to allow partition of the first few MGy of radiation into much lower dose frames.

Fig. S10 shows a plot of first-frame glutamate side chain densities versus the full map densities. The most rapidly damaged side chains (which appear as points furthest above the line) are E125(L,T), which is on the outer surface, and E45(L,T), E57(L,T)/E71(H) and E104(L,T), which are in the central hydro­philic cavity surrounded by lots of water molecules. E315(L,T)/E330(H), although it still has good density in the full map, shows density loss between frames 1 and 2, as shown in Fig. 5 ▸. The least rapidly damaged and well resolved are: E176(L,T)/E191(H), which form salt bridges with arginine/lysine R25(L,T) and K145(L,T) or R189(H); and E297(T), which is stabilized by stacking against histidine H75(T). Fig. 5 ▸ shows examples of glutamic acid side chains that are well ordered initially yet are rapidly damaged by the second frame of the movie. The sensitivity and pattern of radiation damage closely follows that found during electron diffraction of 3D crystals (Hattne *et al.*, 2018 ▸).

From these observations, we conclude that radiation damage in cryoEM continues to be a problem. This may not matter for a wide range of cryoEM investigations where the main goal is to describe the overall structure in rough detail. However, for other research where detailed stereochemistry is critically important, such as for structure-based drug design or description of catalytic mechanism, this will continue to be an important topic for future research, and the best quality maps from movement-free specimens will be necessary. One of the main conclusions of this work is that it would be useful to collect a much larger (10×) dataset so that 3D maps could be calculated with more finely divided movie frames and so a smaller increase in damage between consecutive frames. This is possible since the latest direct electron detectors record the time point of each electron arriving to within less than a millisecond in a several-second overall movie sequence. For a 20 e^−^ Å^−2^ exposure series, this would allow 3D maps to be calculated in steps of less than 0.01 e^−^ Å^−2^ per frame, which corresponds to less than 30 kGy. This would only require more microscope and data processing time to obtain good enough statistics.

### 3.9. Cysteines in human catalase

There are no cysteine residues in either bacterial catalase but there are four in human. Two of these cysteines C232(H) and C460(H) are well resolved, and C377(H) has a clear 50:50 dual conformation. The map in region C393(H)–M394(H) suggests there is some underlying heterogeneity most likely due to inter-subunit clashes between symmetry-related residues. In the X-ray structure 1DGF showed a similar clear 60:40 dual conformation for C377(H), with good density for the other three, with C232(H) strongest and C393(H) weakest just like in the cryoEM map.

### 3.10. Comparison with X-ray structures

The highest resolution catalase X-ray structure is for cat_L at 0.88 Å (Murshudov *et al.*, 2002 ▸). After secondary structure matching (Krissinel & Henrick, 2004 ▸), the core structures of cat_L (9T0M determined by cryoEM and 1GWE determined by X-ray crystallography) are in broad agreement and are well ordered in both maps. The overall X-ray and cryoEM cat_L structures are very similar (overall r.m.s.d. 0.283 Å; Table S2), except that the side chains of Q167, Q153, N173 and Q462 show amide groups in opposite orientations. Careful analysis of the X-ray densities, *B* factors and interactions for those four residues showed that orientations in the X-ray structure were mismodelled.

Q167 in 9T0M is in the most frequent rotamer conformation (mt-30; Lovell *et al.*, 1999 ▸) and its amide orientation appears consistent with the hydrogen bonding environment of a well ordered water molecule common to both structures. In 9T0M, this water molecule HOH834(L) is hydrogen bonded to O of S152, O of K154, N of D163 and NE2 of Q167, while in 1GWE the equivalent HOH2290 would have OE2 of Q167 as its fourth hydrogen-bonding partner.

Q153 in 9T0M is in a more frequently observed rotamer conformation than in 1GWE (15% and 3%, respectively), but this side chain faces the water channel so both orientations are plausible.

N173 shows the opposite amide orientations in 9T0M and 1GWE. In 9T0M, ND2 of N173 is hydrogen bonded with O and OD1 of D169. Therefore this ND2 cannot be exchanged with OD1.

Q462 in 9T0M has NE2 hydrogen bonded to O of L454, but in 1GWE OE1 at this position clashes with O of L454.

Some surface side chains show flexibility in both X-ray and cryoEM maps, characterized by multiple conformations or high *B* factors in the X-ray structure and fuzzy density in the cryoEM map. The wrapping loop between subunits A-B and C-D is identical. The same complicated regions on the tetramer twofold axes at L105-G106 and at Y378 are also observed in the cryoEM map. At regions of tangled density with more than one conformation, the advantage of cryoEM is that different attempts at model building do not alter the observed map. At places where the side chains or backbone from one subunit are on one of the twofold axes, the equivalent symmetry-related residue cannot have the same conformation, so the maps show the average of the two states. There are three glutamine side chains, Q167, Q153 and Q462, in cat_L where a different choice of γδ side chain flip was made during model building, as discussed above. While the two side chain conformations are almost indistinguishable in the maps, the correct choice will be important for form-factor determination. All valine, leucine and isoleucine rotamers are identical.

The two main differences between the X-ray and the cryoEM structures were (i) the absence of NADPH in the X-ray structure, where sulfate ions from the 1.4 *M* ammonium sulfate used in the crystallization displaces the NADPH, and (ii) the presence of the 16 C-terminal residues E488-I503 in the X-ray structure and not in the cryoEM structure. The N-terminus was better ordered in the cryoEM map, and the C-terminus was better ordered in the X-ray map. The visibility of the C-terminus in the X-ray structure, which was slightly more disordered than the rest of the molecule, may have been caused either by stabilization due to crystal contacts or by the 30× higher ionic strength used in crystallization than for cryoEM. Either or both possible explanations will need to be investigated experimentally. Side chains R162 and D163 made contact with the ordered C-terminus in the X-ray map, yet both showed the same clear density in both X-ray and cryoEM maps. Similarly, the side chains that interact with NADPH were well ordered in the X-ray map with water molecules filling the space vacated by NADPH.

Comparing the human erythrocyte catalase structure cat_H with the X-ray structure 1DGF (Putnam *et al.*, 2000 ▸) indicated the presence of NADPH in only two of the four subunits in their orthorhombic crystal form, whereas the cryoEM map showed full occupancy in the *D*2 symmetry-averaged map. The overall r.m.s.d. between the cryoEM structure and the X-ray structure of the NADPH-occupied subunit is 0.202 Å (Table S2). Putnam *et al.* (2000 ▸) suggest that the two empty NADPH sites are partly blocked by crystal contacts which also cause F198(H), H305(H) and V450(H) to move in the absence of NADPH. F198(H) and H305(H) both rotate 120° about their αβ bond.

In *Proteus mirabilis*, Gouet *et al.* (1995 ▸) also specifically compared the structure without and with NADPH in a clade 3 catalase obtained by soaking ligand-free crystals in NADPH solution. They found the equivalent residue to H305(H) rotates 50° when NADPH is absent but observed no change in the F198(H) equivalent, strongly suggesting that crystal contacts in cat_H perturb the cat_H NADPH binding site.

## 4. Conclusion

The three catalase structures we have obtained show many interesting features. Some surface loops showed conformations where equivalent residues in the structures obtained by X-ray crystallography were no longer influenced by crystal contacts. The cryoEM maps showed a spectrum of radiation damage to amino acid side chains, predominantly glutamic acid. As generally accepted, buried side chains or those that are in strong salt bridges have the best resolved density and the least radiation damage, whereas many of those exposed on the surface or to the solvent environment showed weak or weaker density. This was where most radiation damage and conformational flexibility occurred. CryoEM structure determinations, including those in this paper, are typically affected by greater levels of radiation damage than occur in typical X-ray crystal structure determinations, because the energy deposited is higher. There is therefore a strong argument to collect a significantly larger dataset for at least one structure to allow dose fractionation into finer movie frames, which would permit observation of the undamaged structure by cryoEM in plunge-frozen single-particle samples. This would then open the possibility of being able to determine the charge state of residues such as aspartate, glutamate and histidine, a long-term goal that has been plagued with justifiable concern about radiation damage. Metalloenzymes sometimes show extreme sensitivity to X-ray- or electron-induced metal reduction, which is a type of radiation damage, and this could also be investigated with a larger dataset.

## Supplementary Material

Supplementary tables and figures. DOI: 10.1107/S2052252526008845/rq5021sup1.pdf

PDB reference: human erythrocyte catalase, 9T0K

PDB reference: *Rhizobium radiobacter* catalase, 9T0L

PDB reference: *Micrococcus luteus* catalase, 9T0M

EMDB reference: human erythrocyte catalase, EMD-55403

EMDB reference: *Rhizobium radiobacter* catalase, EMD-55404

EMDB reference: *Micrococcus luteus* catalase, EMD-55405

## Figures and Tables

**Figure 1:**
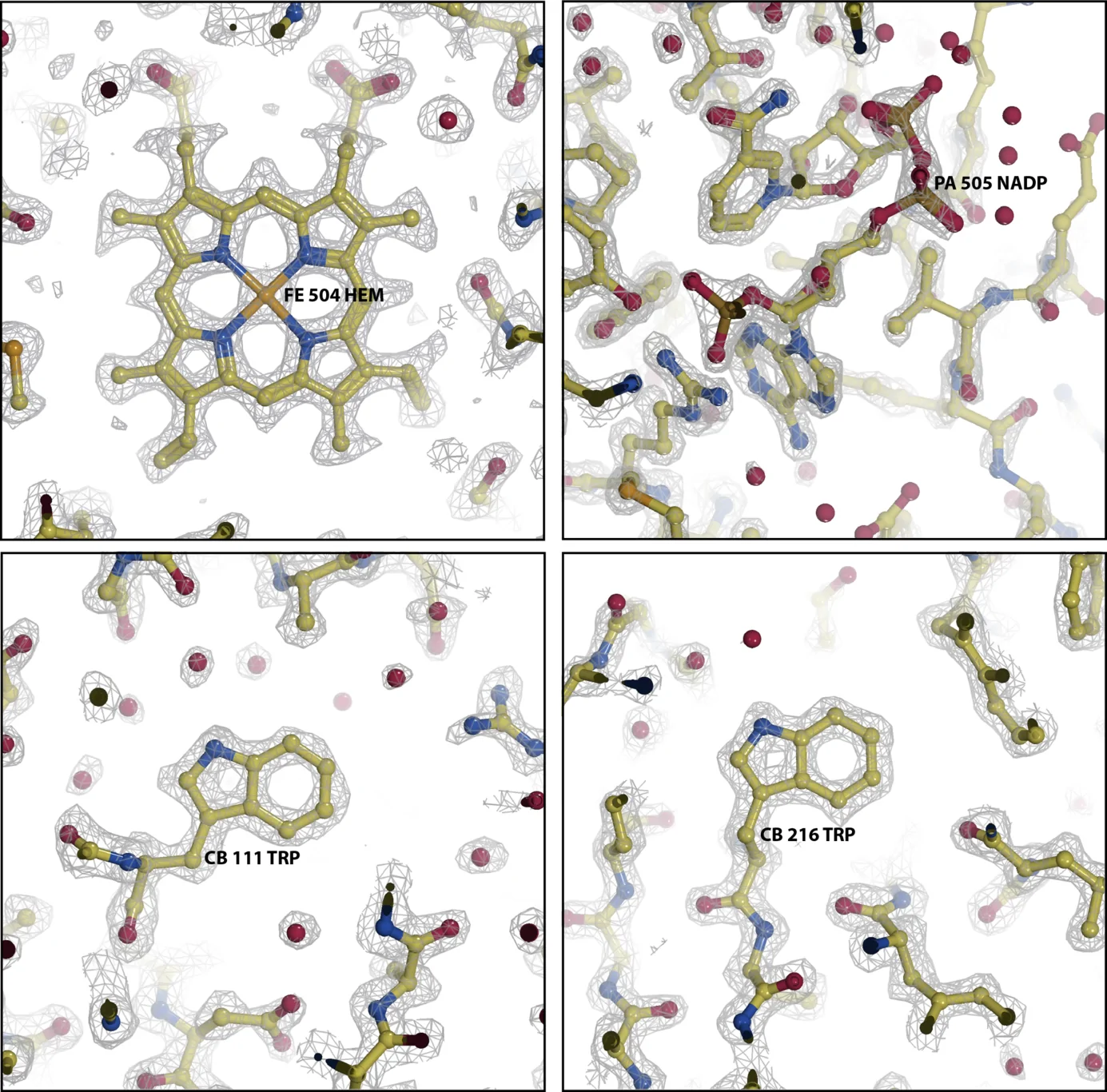
Potential distribution in cat_T cryoEM map with model superimposed: (*a*) haem, (*b*) NADPH, (*c*) Trp-111, (*d*) Trp-216. Hydrogen atoms were not explicitly refined and are not shown, yet these map regions all show clear evidence for hydrogen-atom densities. The contour threshold used was chosen to illustrate the densities for the haem, nicotinamide and tryptophan, so that the holes in each ring structure are clear, as expected at a resolution of 1.7 Å. A few of the water molecules in the model have lower occupancy and do not show their accompanying density at this contour threshold.

**Figure 2:**
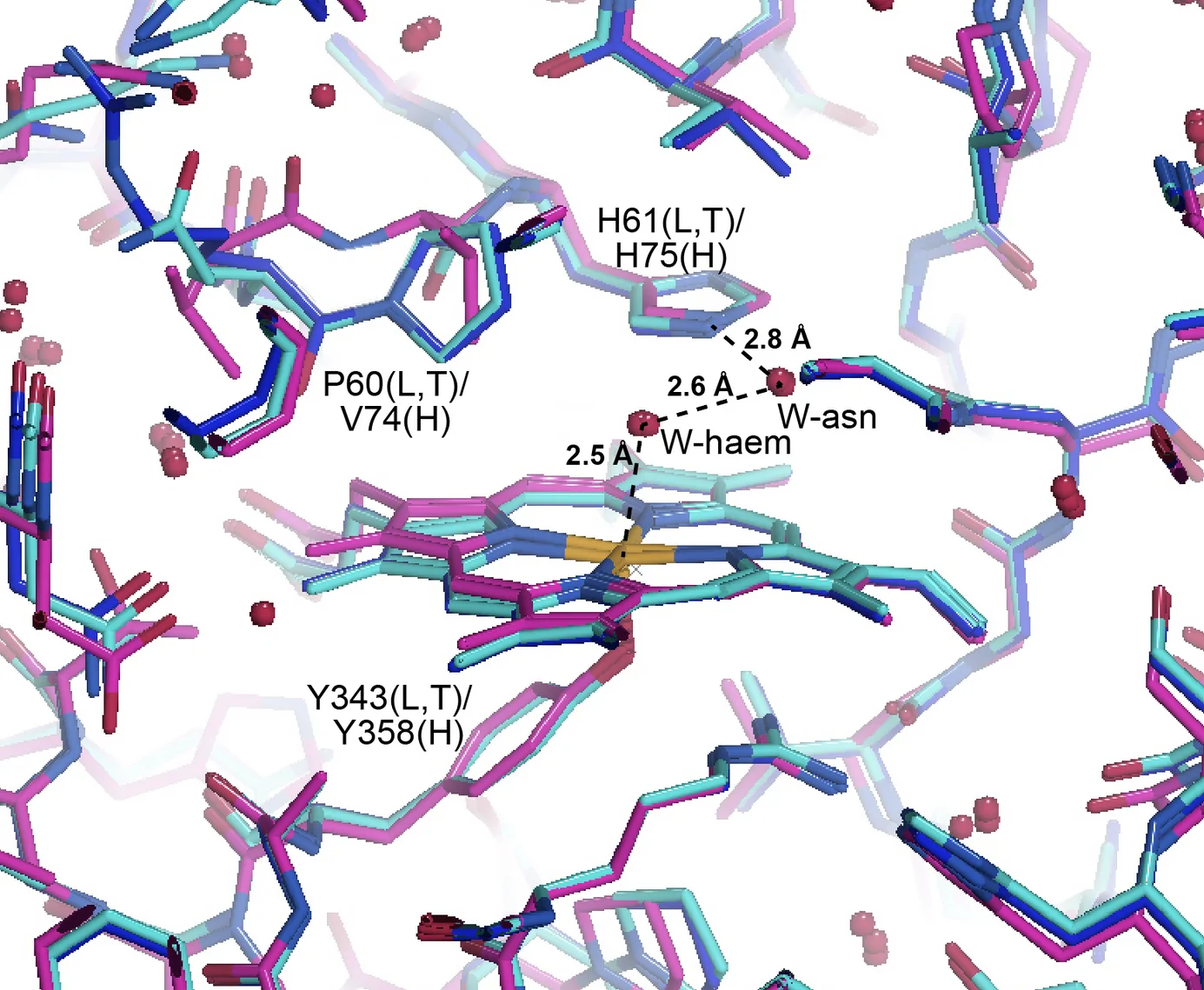
Side-by-side haem environments. Cat_T dark blue, cat_L light blue, cat_H pink. The close similarity of cat_L and cat_T and the slight tilt of the cat_H haem are clear. The distances between the two haem-pocket water molecules and the active-site residues are labelled.

**Figure 3:**
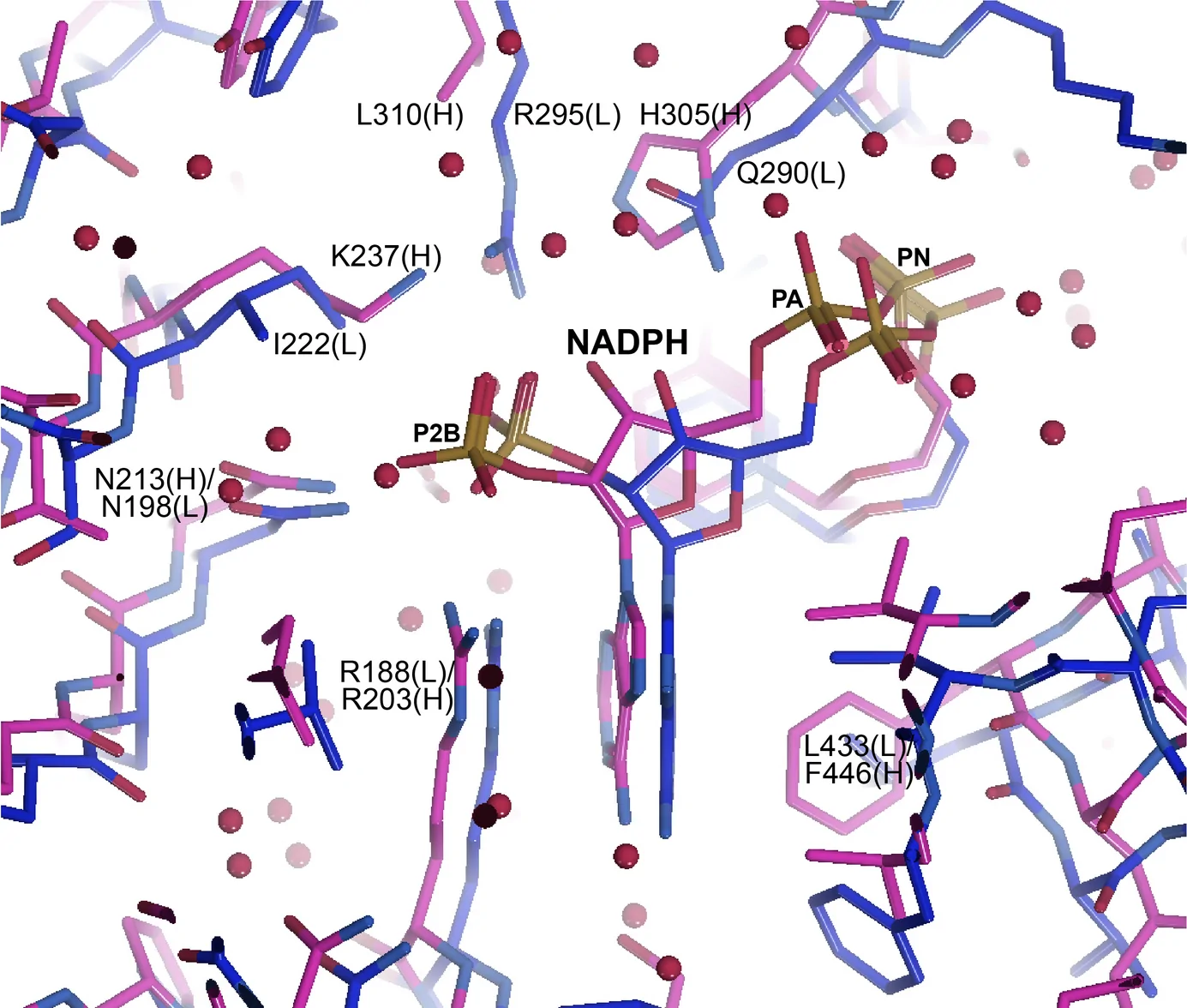
Side-by-side NADPH environments. Cat_T blue, cat_H pink. Although the NADPH conformation itself is very similar, the surrounding residues and water molecules have significant differences. Of the six surrounding residues that are labelled, four have significantly different identities. Other residues near NADPH are also discussed in the text; H222(H) is in front of this slice with its NE2 forming a 3.04 Å hydrogen bond with OX3 of NADPH; Y183(T,L)/F198(H) is behind the slice and 3.4 Å from NADPH.

**Figure 4:**
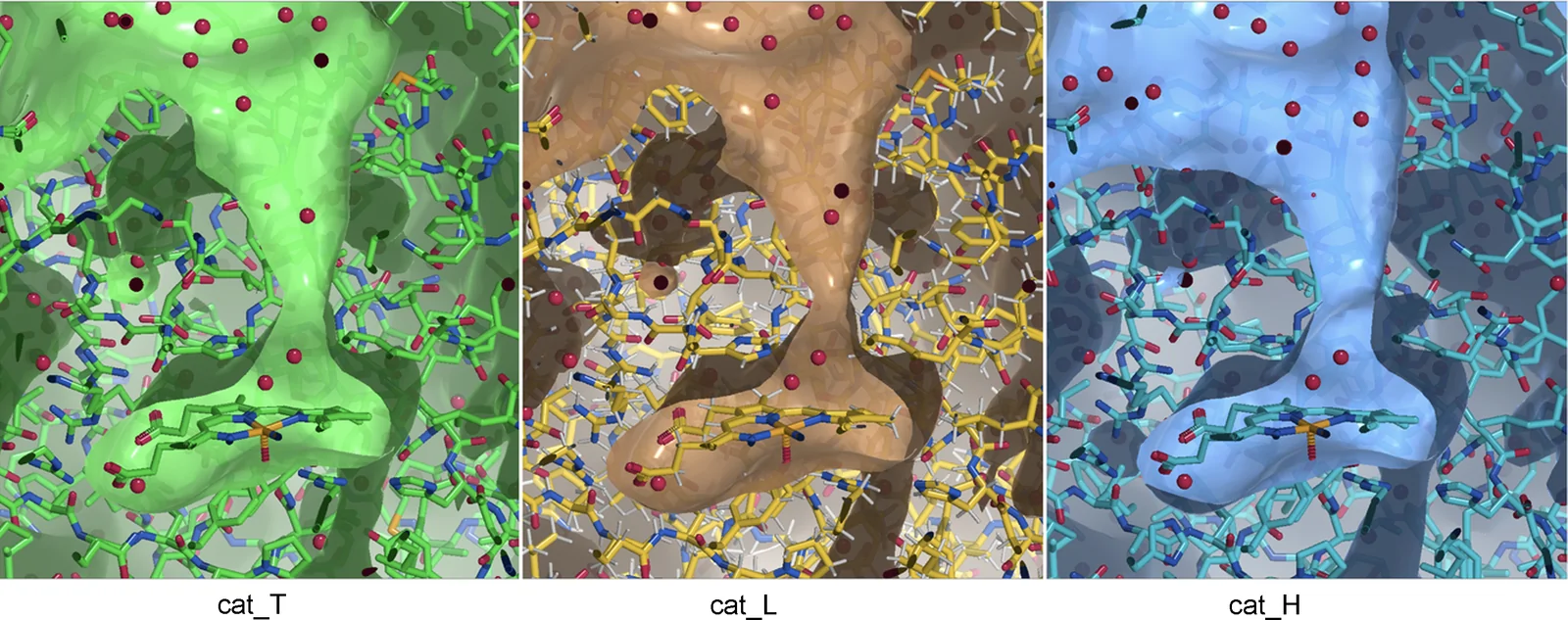
Side-by-side images of the aqueous channel in cat_T, cat_L and cat_H through which H_2_O_2_ gains access to the active site, leading from the haem group to the molecular surface at the top. The Gaussian surface was created from the coordinates without the haem and water molecules. The atomic model includes both. The water molecules are shown as red spheres, with the two at the haem iron and the active-site histidine/asparagine in almost identical positions. The ordered water molecules higher in the channel have slightly different locations, and less well ordered water fills the remainder of the channels.

**Figure 5:**
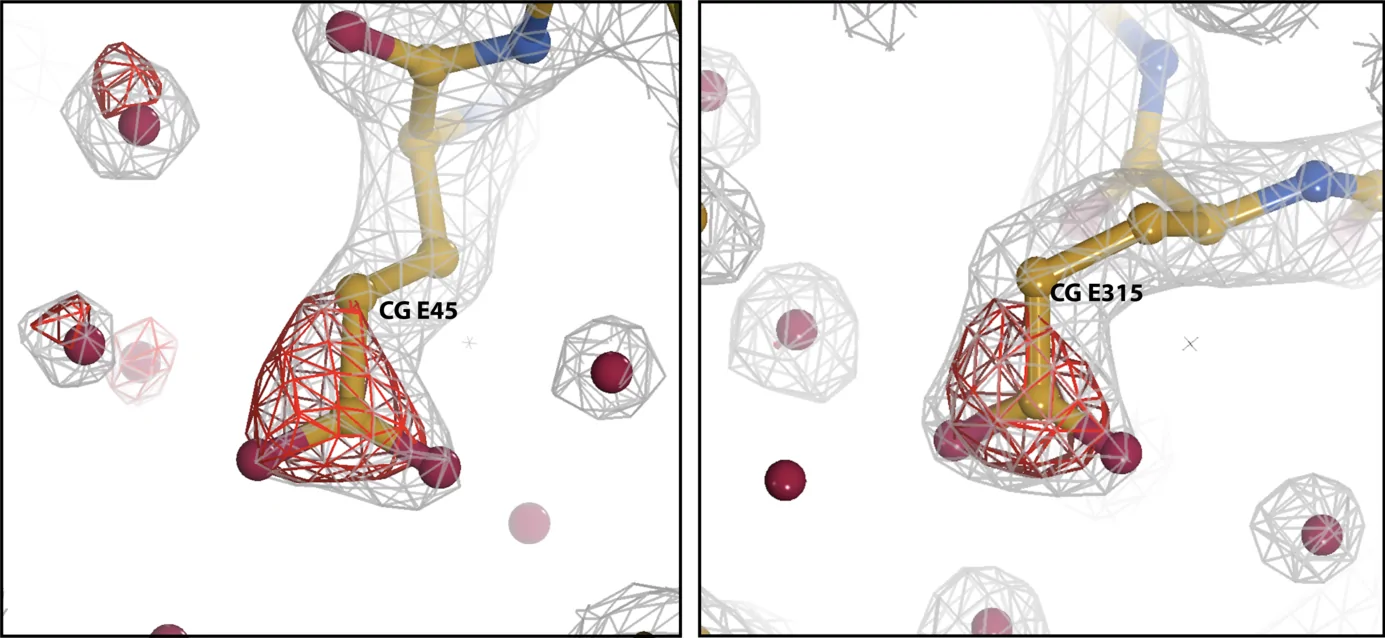
Examples of radiation damage to two glutamic acid side chains, E45 and E315, in cat_T. The grey mesh shows the full map. The red mesh shows the difference (loss of mass) between the 3D maps calculated between the first and second frames of the data collection movies, corresponding to a dose of 1.8 e^−^ Å^−2^.

**Table 1:** X-ray crystallography clade 3 catalase structures in the PDB with resolution better than 2.0 Å

PDB	Name/species/tissue	Resolution (Å)	Date deposited
1DGF, 1DGG	*Homo sapiens*, erythrocyte	1.5, 1.8	1999
1GWE, 1GWF, 1GWH	*Micrococcus lysodeikticus*	0.88, 1.5, 1.96	2002
1QWL, 2A9E, 1QWM, 2IQF, 9NH3	*Helicobacter pylori*	1.6, 1.76, 1.6, 1.86, 1.87	2003, 2005, 2006, 2025
2ISA	*Vibrio salmonicida*	1.97	2006
3RE8, 3RGP, 3RGS, 6PO0, 6PM7	*Bos taurus*, beef liver	1.9, 1.88, 1.99, 1.75, 1.85	2012, 2019
1M85	*Proteus mirabilis*	2.0	2002
4B7G, 4B7H, 4B7F	*Corynebacterium glutamicum*	1.9, 1.39, 1.76	2012
6RJR	*Kluyveromyces lactis*	1.895	2019

**Table 2:** Statistics of data collection, PDB and EMDB deposition

Property	cat_H	cat_L	cat_T
Species name	*Homo sapiens*	*Micrococcus luteus*	*Rhizobium radiobacter*
Number of movies	24991	22747	35543
Initial number of particles picked	2187815	1571217	2071946
Final number of particles	1070801	261172	990333
Maximum beam tilt (mrad)	0.08	0.14	0.45–0.76
Resolution (Å)	1.87	1.92	1.73
Efficiency of orientation distribution, *E*_od_	0.72	0.75	0.75
*Q*-score	0.802	0.800	0.838

Visible residues in 3D cryoEM map	Ser4-Glu503	Thr7-His487	Gly10-Gln489
Number of water molecules	397	422	425
Comparable visible residues in X-ray map	Arg5-Asn501	Thr6-Ile503	—
Comparable X-ray structure PDB identification	1DGF, 1DGG	1GWE, 1GWH	—
			
PDB identification number – coordinates	9T0K	9T0M	9T0L
EMDB identification number – cryoEM maps	EMD-55403	EMD-55405	EMD-55404
