# Supplementary material for: Comparison of human and bacterial monofunctional catalase structures obtained by electron cryomicroscopy
Source: IUCrJ. 2026 Nov 3;13(Pt 6):807–18. doi: 10.1107/S2052252526008845 (PMC13763893; doi:10.1107/S2052252526008845)
Supplement: Supplementary file 1 [file m-13-00807-sup1.pdf]

IUCrJ

Volume 13 (2026)

Supporting information for article:

Comparison of human and bacterial monofunctional catalase structures obtained by electron cryomicroscopy

Daria Slowik, Jade Li, Hugh Wilson, Alexander Shtyrov, Shaoxia Chen, Greg McMullan, Christopher J. Russo, Garib Murshudov and Richard Henderson

	cat_H	cat_L	cat_T
cat_H	100.00 %	44.06 %	47.33 %
cat_L	44.06 %	100.00 %	68.78 %
cat_T	47.33 %	68.78 %	100.00 %

Table S1 Percent sequence identity (38% for all three catalase sequences)

PDB	Cat_H 9T0K EM	Cat_L 9T0M EM	Cat_T 9T0L EM	Cat_H 1DGF X-ray	Cat_L 1GWE X-ray
Cat_H 9T0K EM	0.0	0.972 (463)	1.014 (466)	0.202 (497)	1.014 (465)
Cat_L 9T0M EM		0.0	0.410 (478)	1.014 (465)	0.283 (481)
Cat_T 9T0L EM			0.0	1.034 (466)	0.471 (478)
Cat_H 1DGF X-ray				0.0	1.014 (465)
Cat_L 1GWE X-ray					0.0

Table S2 RMSD (Å) between Cα of aligned residues (number aligned) using secondary-structure matching (SSM) from Krissinel & Henrick (2004)

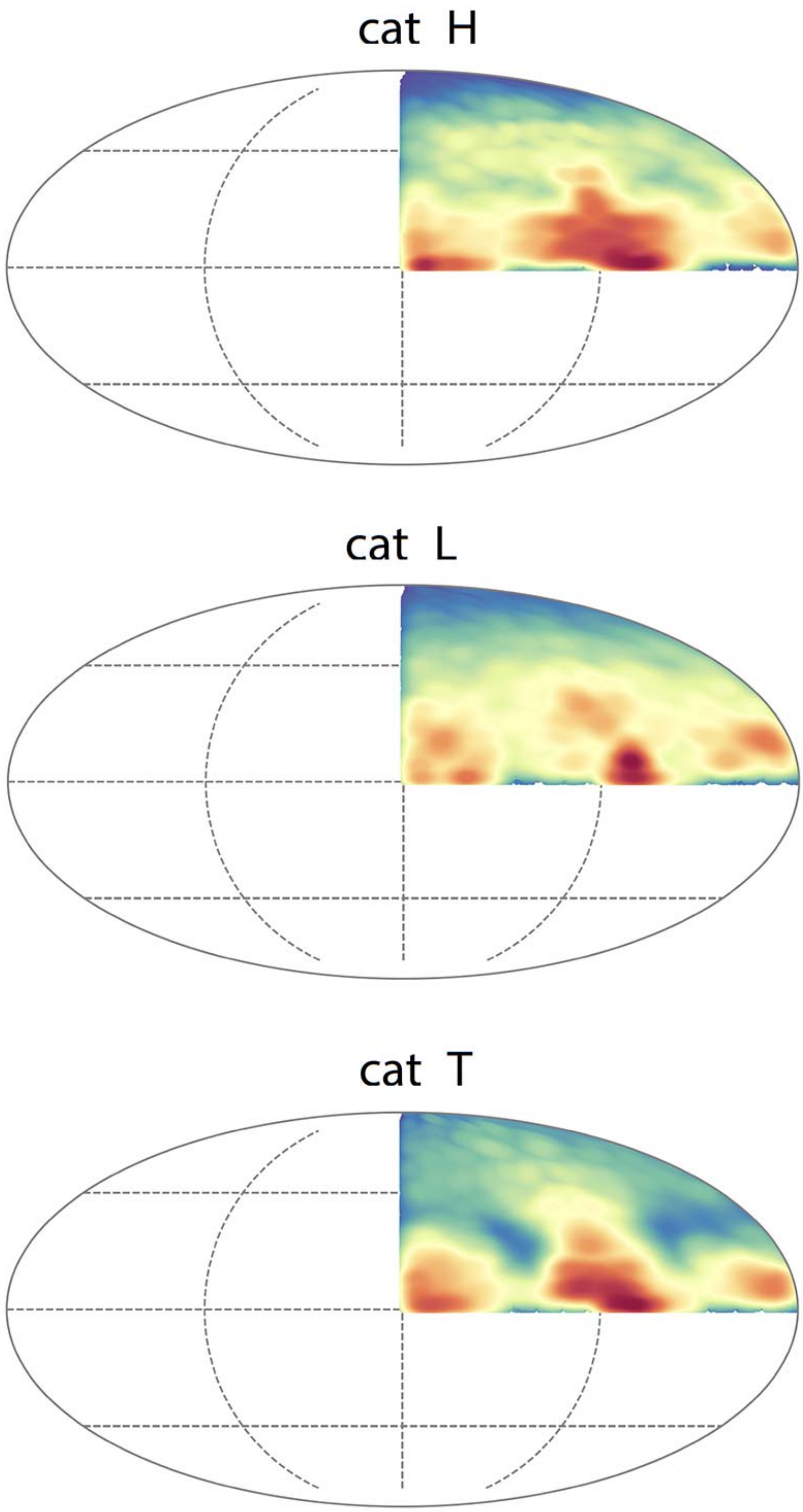

Figure S1 Orientation distributions. Orientation distributions for the three datasets using the *Eod* program from (Naydenova & Russo, 2017).

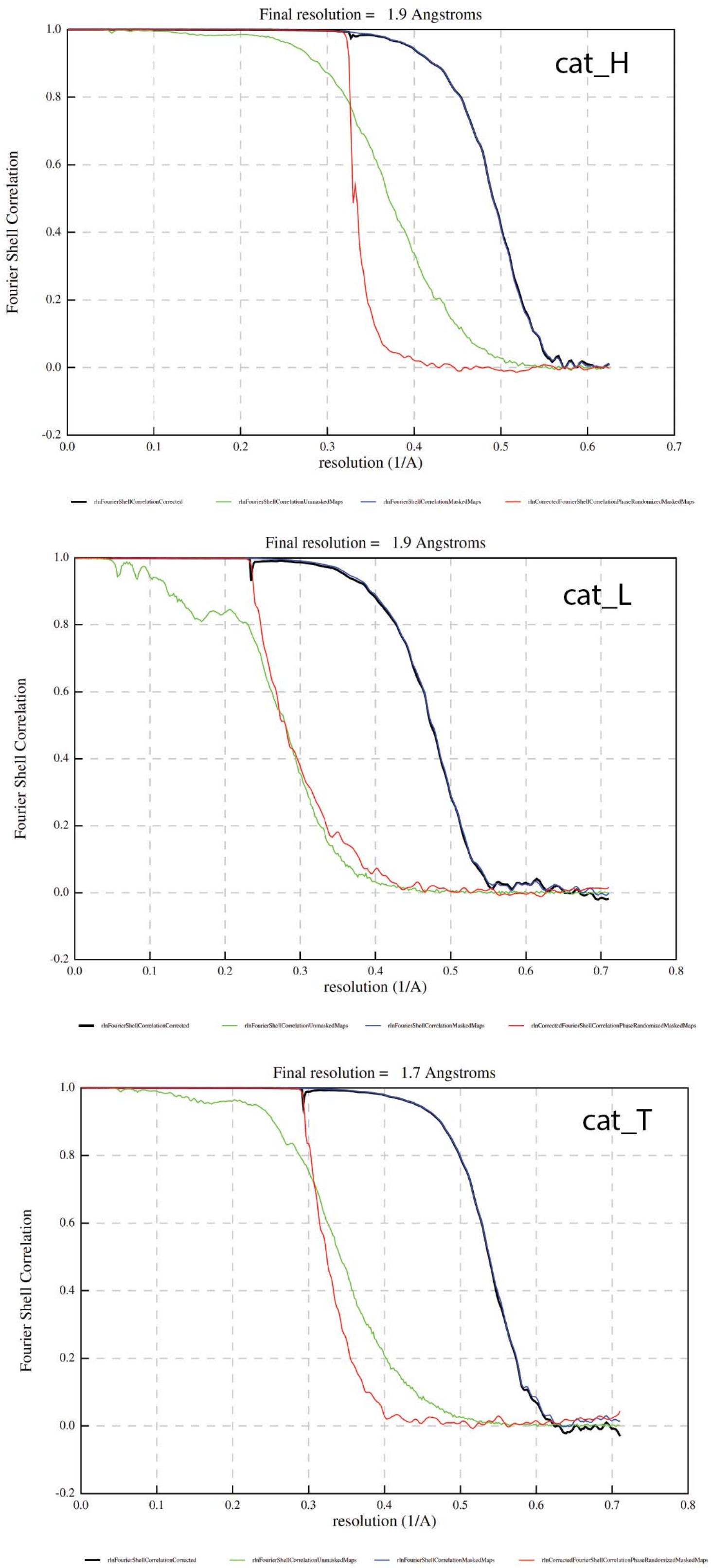

Figure S2 FSC plots for the three maps from the RELION postprocessing step.

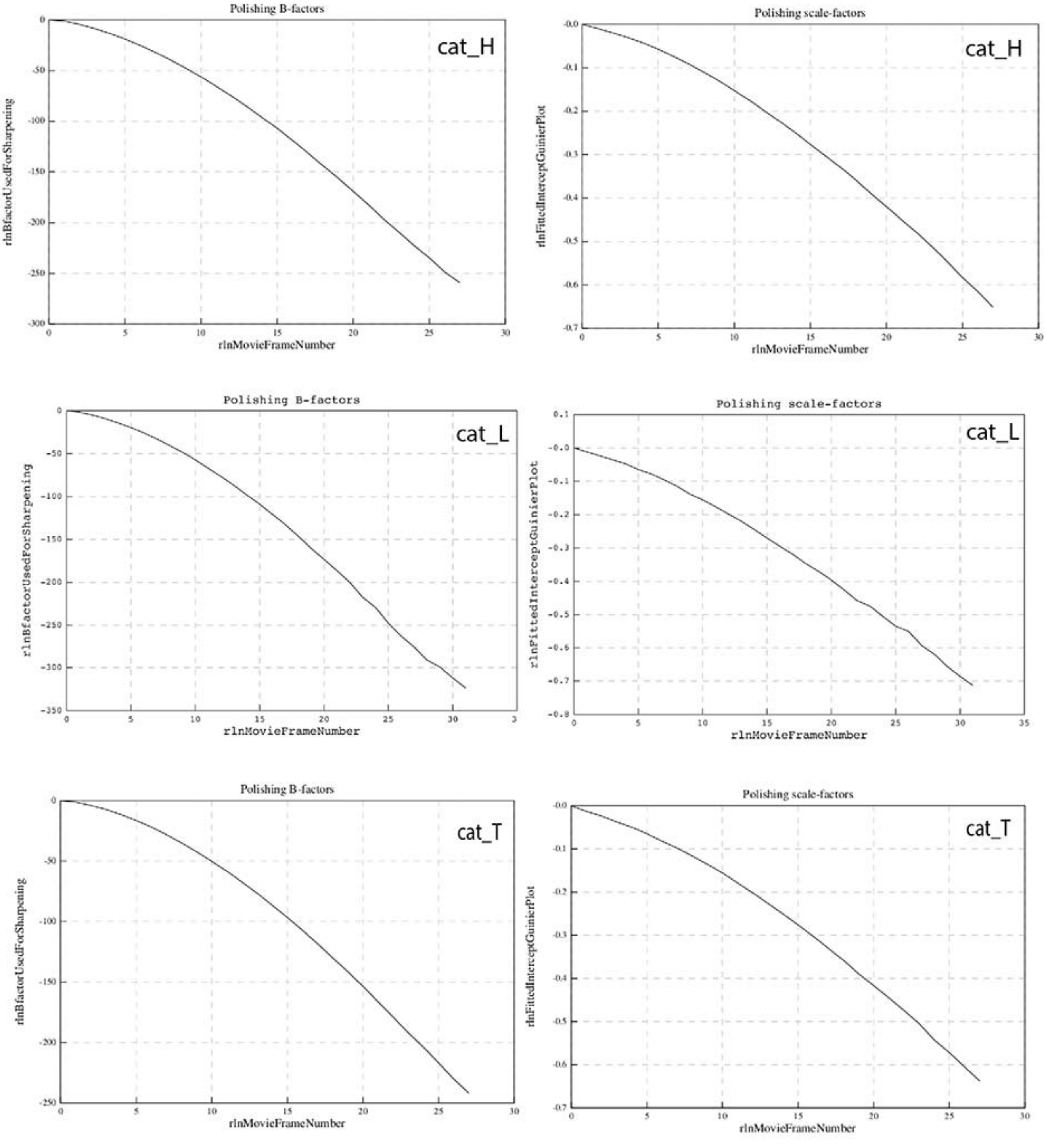

Figure S3 B-factor and intercept plots for movie frames from RELION polishing step.

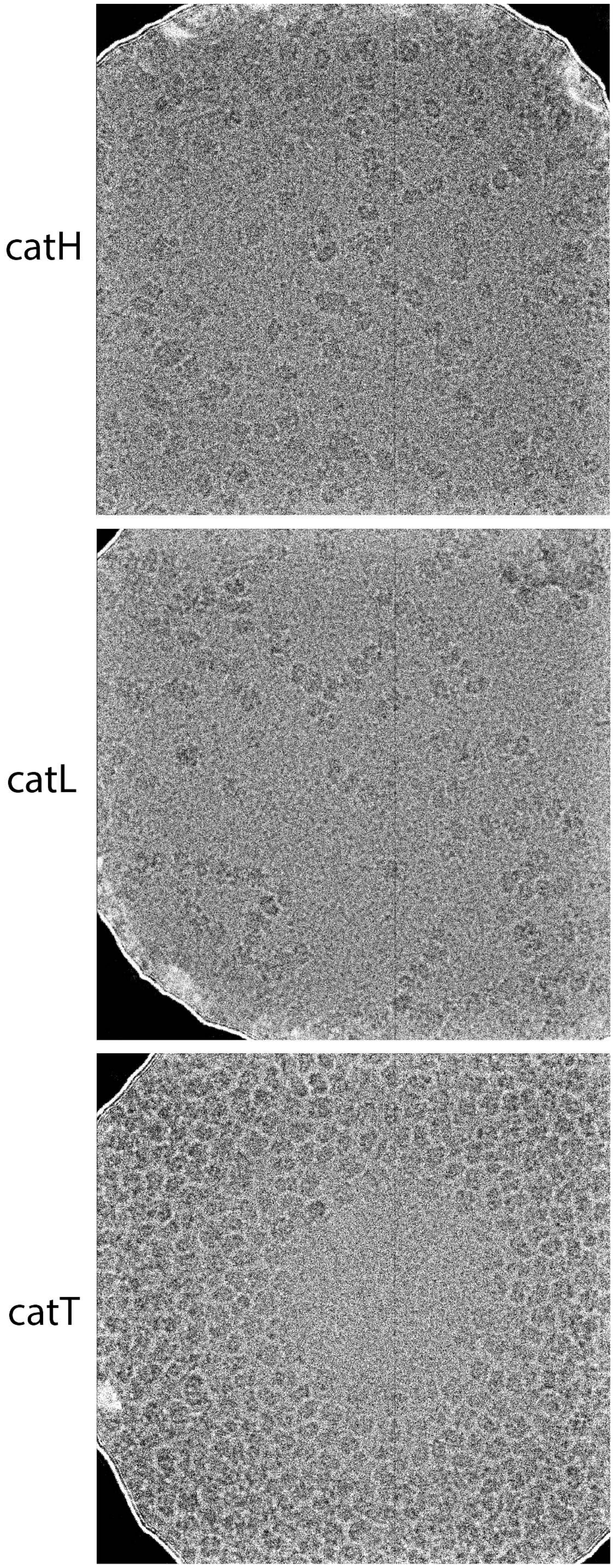

Figure S4 Examples of a micrograph from each of the three datasets. Each image shows the full 4096 x 4096 area from the dose-weighted summation following motion-correction in RELION. Grey levels have been

selected so that the particle distributions can be seen. The edges of the ~300 nm diameter holes in the HexAuFoil gold grids appear in some corners of the images, since the field of view is 210 nm. The particle distribution is frequently denser at the edges of the holes where the ice is thicker, cat_L shows a tendency to forms strings of particles and cat_T tended to form thicker areas despite the presence of CHAPSO detergent. The vertical line in the same place on all three images is a column defect in the detector.

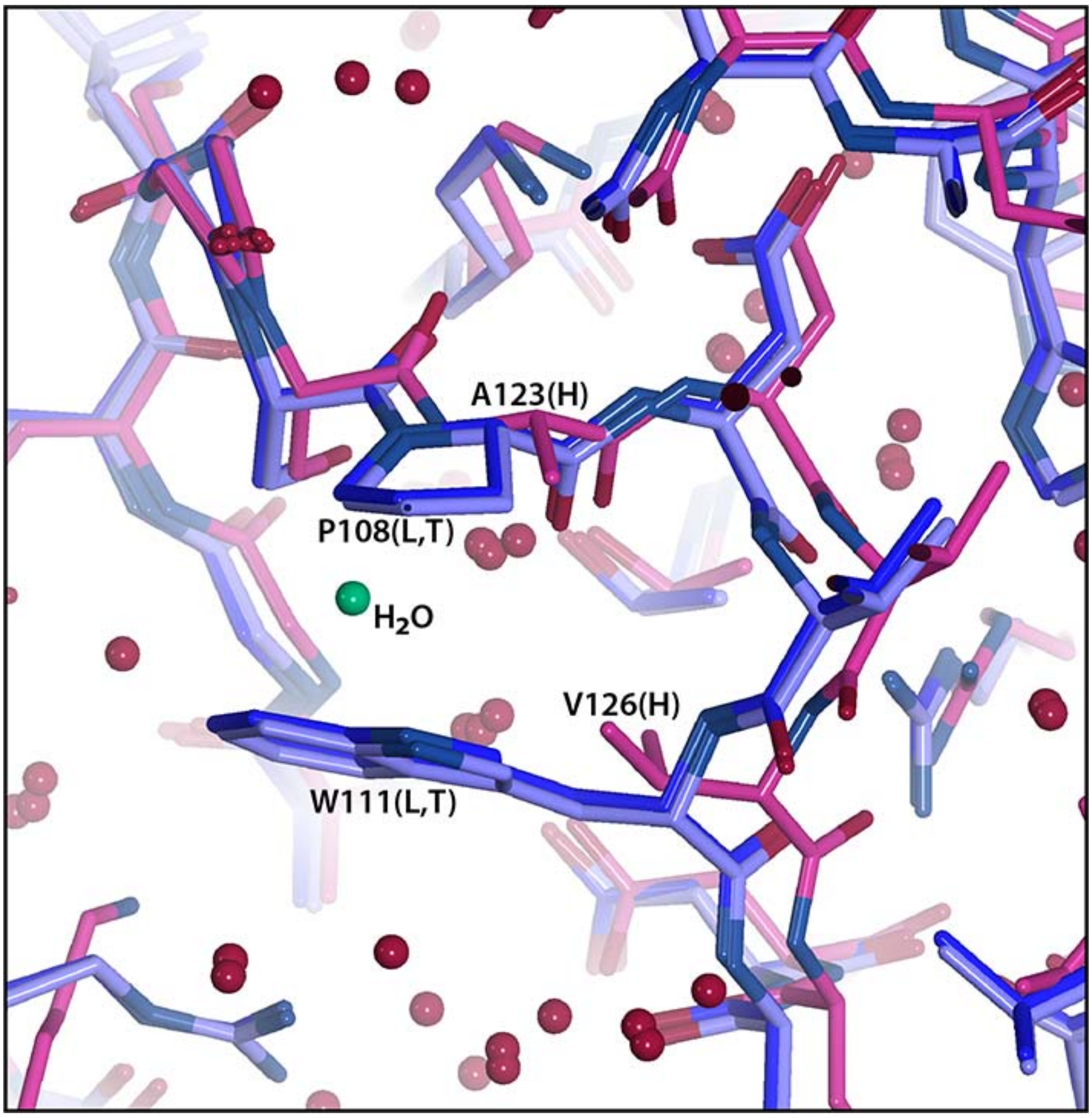

Figure S5 Superimposed region of the structures in which P108(L,T) is replaced by A123(H) and W111(L,T) is replaced by V126(H), creating a cavity in human catalase, which is filled by an additional water molecule, coloured green in the figure. Other water molecules that are in similar positions are shown in the standard Coot red colour.

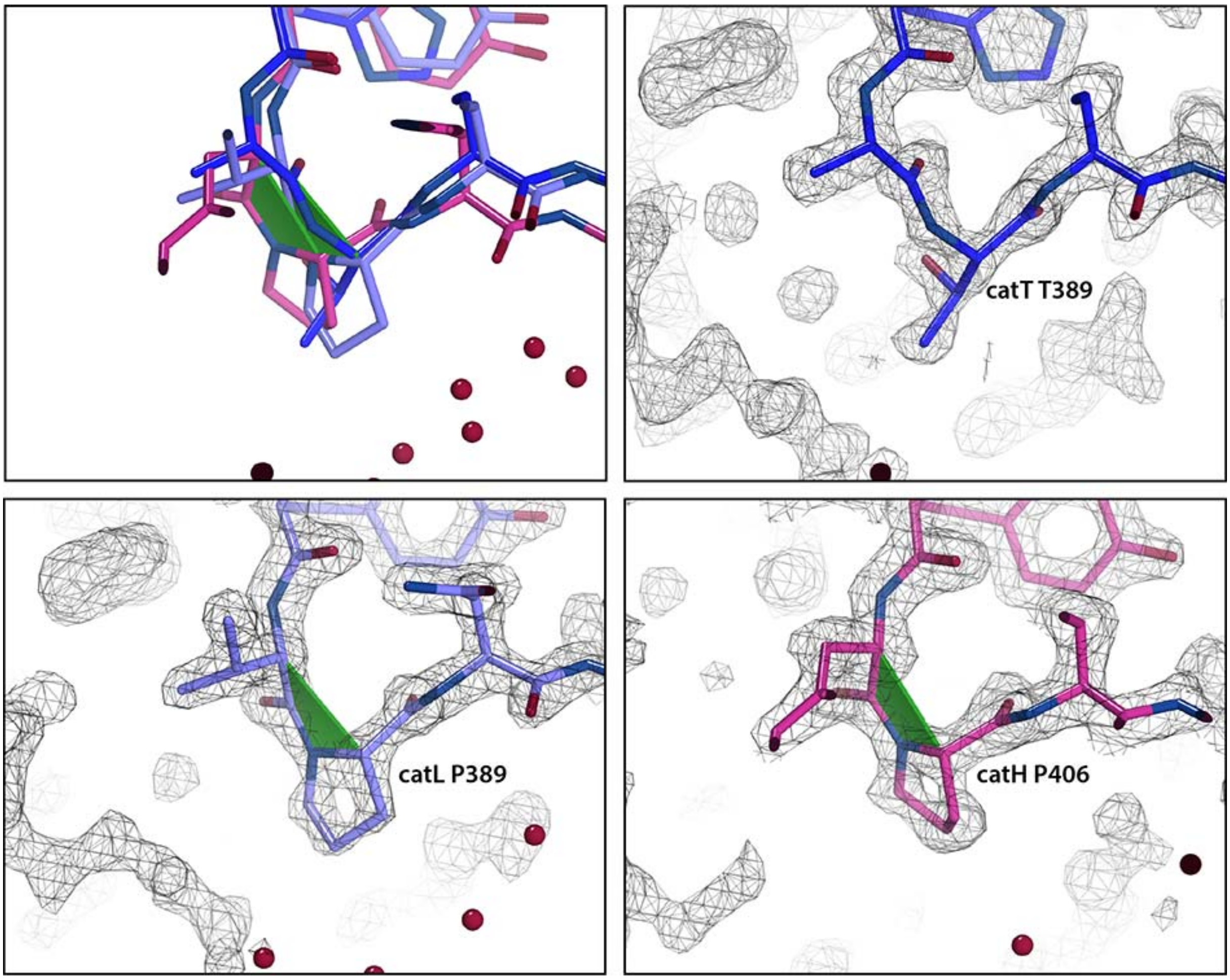

Figure S6 Region in which cis-proline residues, P389 in cat_L and P406 in cat_H are replaced by T389 in cat_T. The first panel shows the superimposed models with no maps. The other three panels show the individual maps plus models. Only the model atoms for the stretch of five amino acids on each side are shown; the densities in the cat_T and cat_L panels without model atoms is from a symmetry-related subunit.

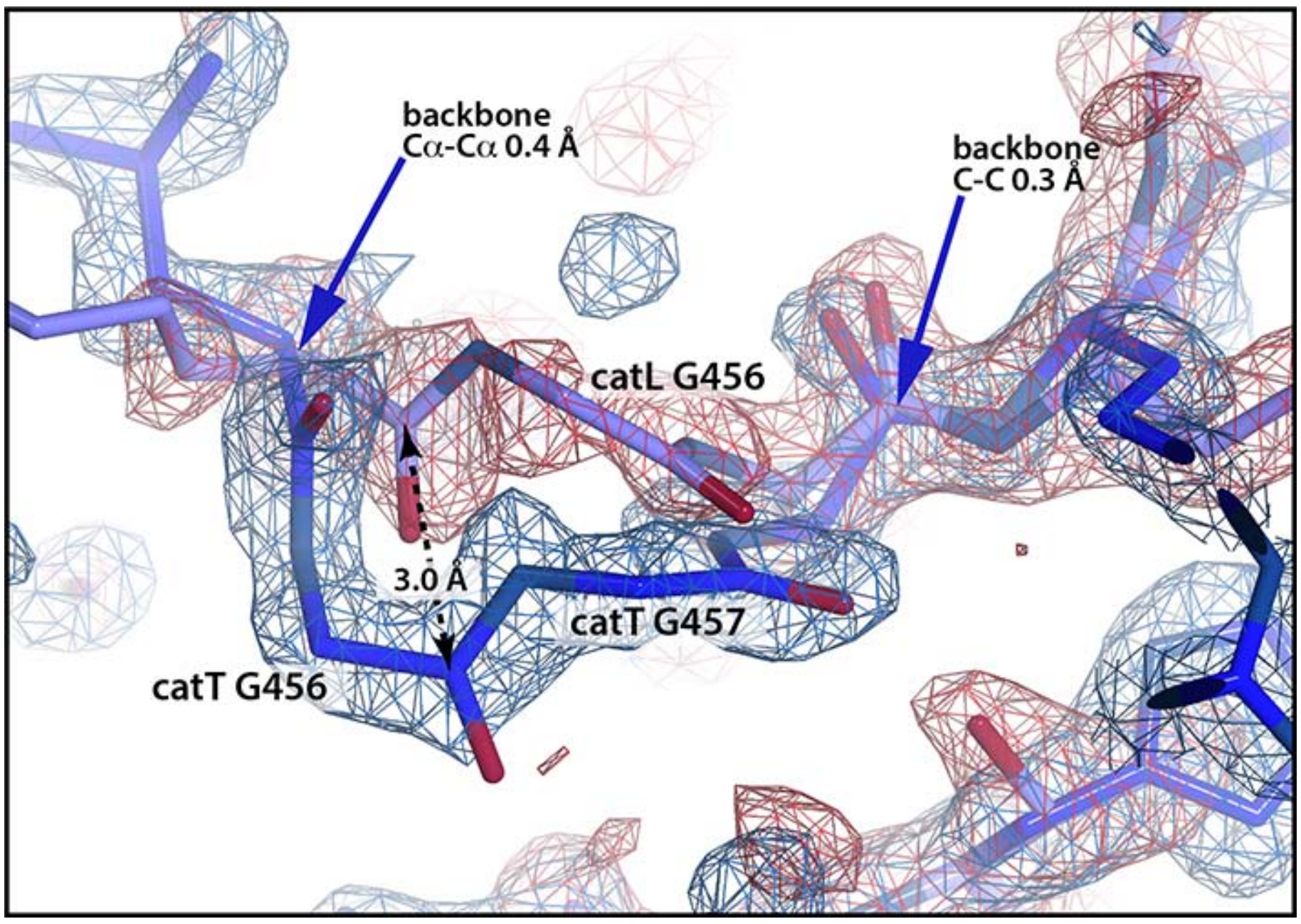

Figure S7 Comparison of the surface loops in which a single glycine is inserted into cat_T relative to cat_L. G456-G457 in cat_T is substituted by a single G456 in cat_L. The backbone registration before and after the insertion is within 0.4 Å for the Cα of residue 455 and 0.3 Å for the carbonyl carbon of the following residue.

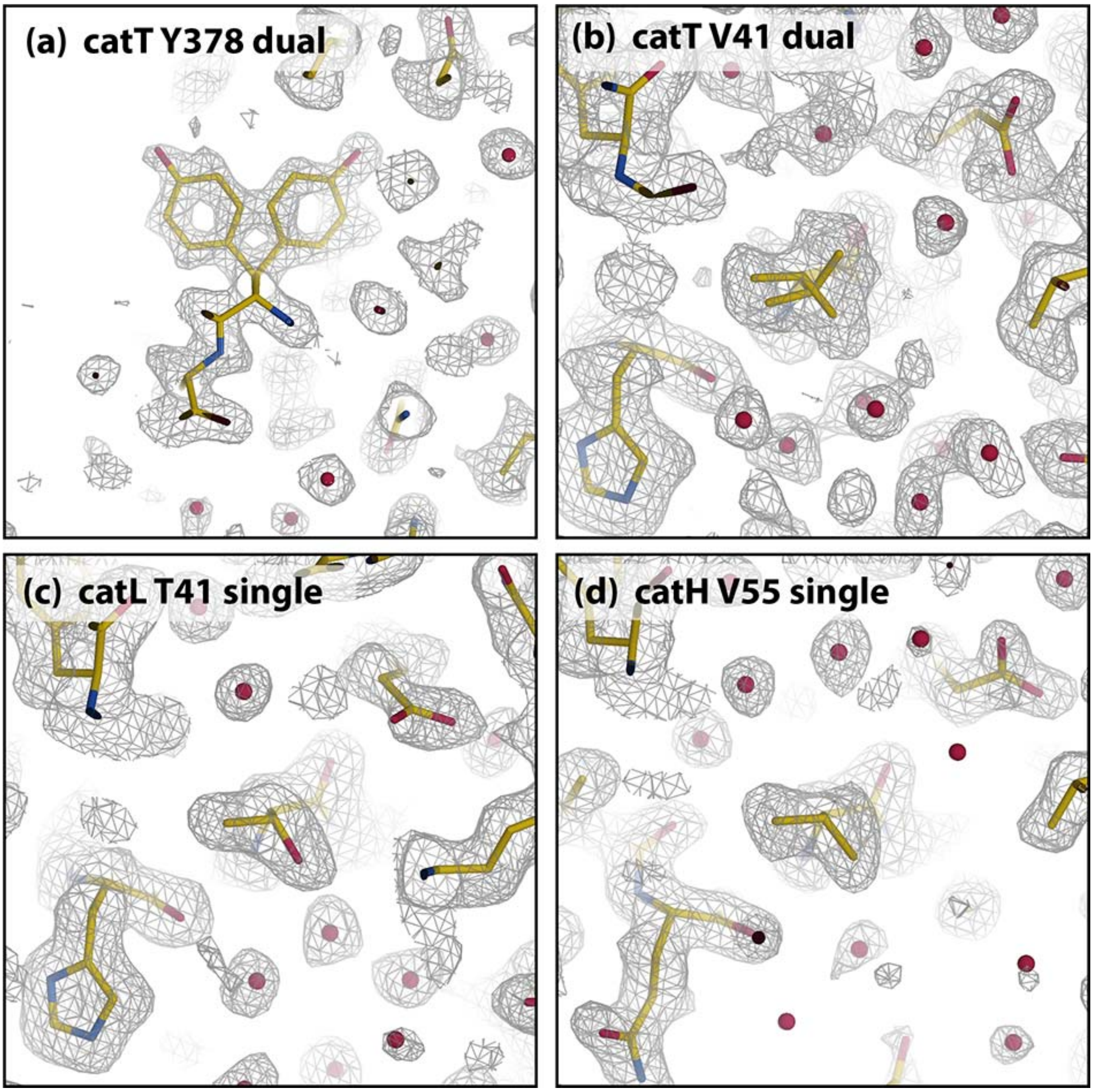

Figure S8 Examples of residues with alternate conformations. In (a) the density and model for Y378 in cat_T is shown, which is adjacent to one of the molecular twofold axes. The two residues in adjacent subunits flip-flop together, so the occupancy of the two conformations is exactly 50:50. The same alternate conformation is found in cat_L but does not occur in the corresponding M394 in cat_H. In (b), V41(T) has a clear alternate conformation, whereas (c,d) only a single conformation is present in the homologous T41(L) and V55(H).

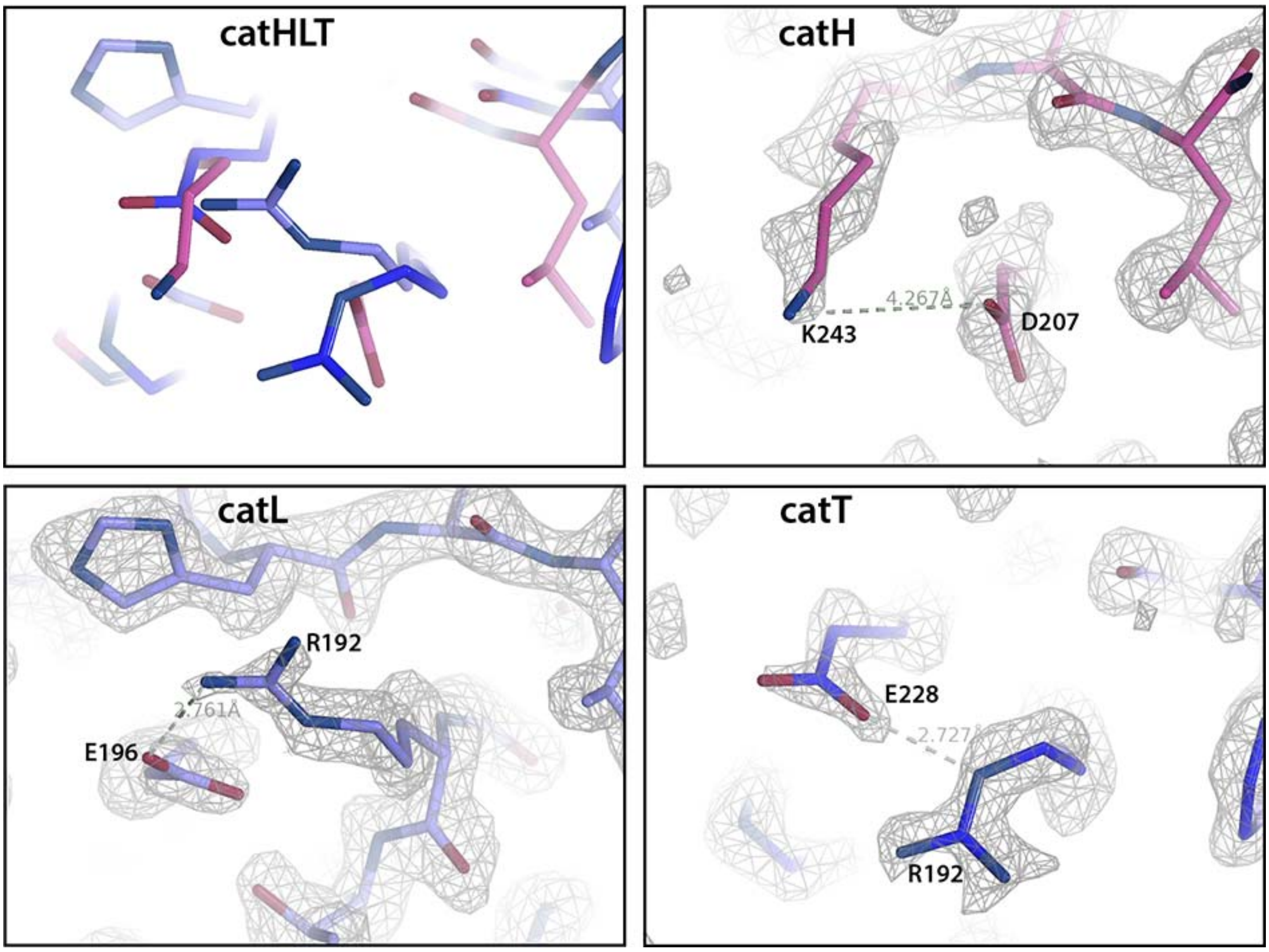

Figure S9 Example of a salt bridge that is different in the three structures. The first panel shows all three models without any map density. The remaining three panels show the individual models plus maps, with good density for all side-chains. The colouring for cat_H is pink, cat_L is pale blue and cat_T is dark blue; cat_H has juxtaposed charges D207 and K243 with Y260 hydrogen-bonded to D207; cat_L has an ion pair between R192 and E196; cat_T has an ion pair between R192 and E228 but with a completely different γδ-rotamer for the side-chain of R192.

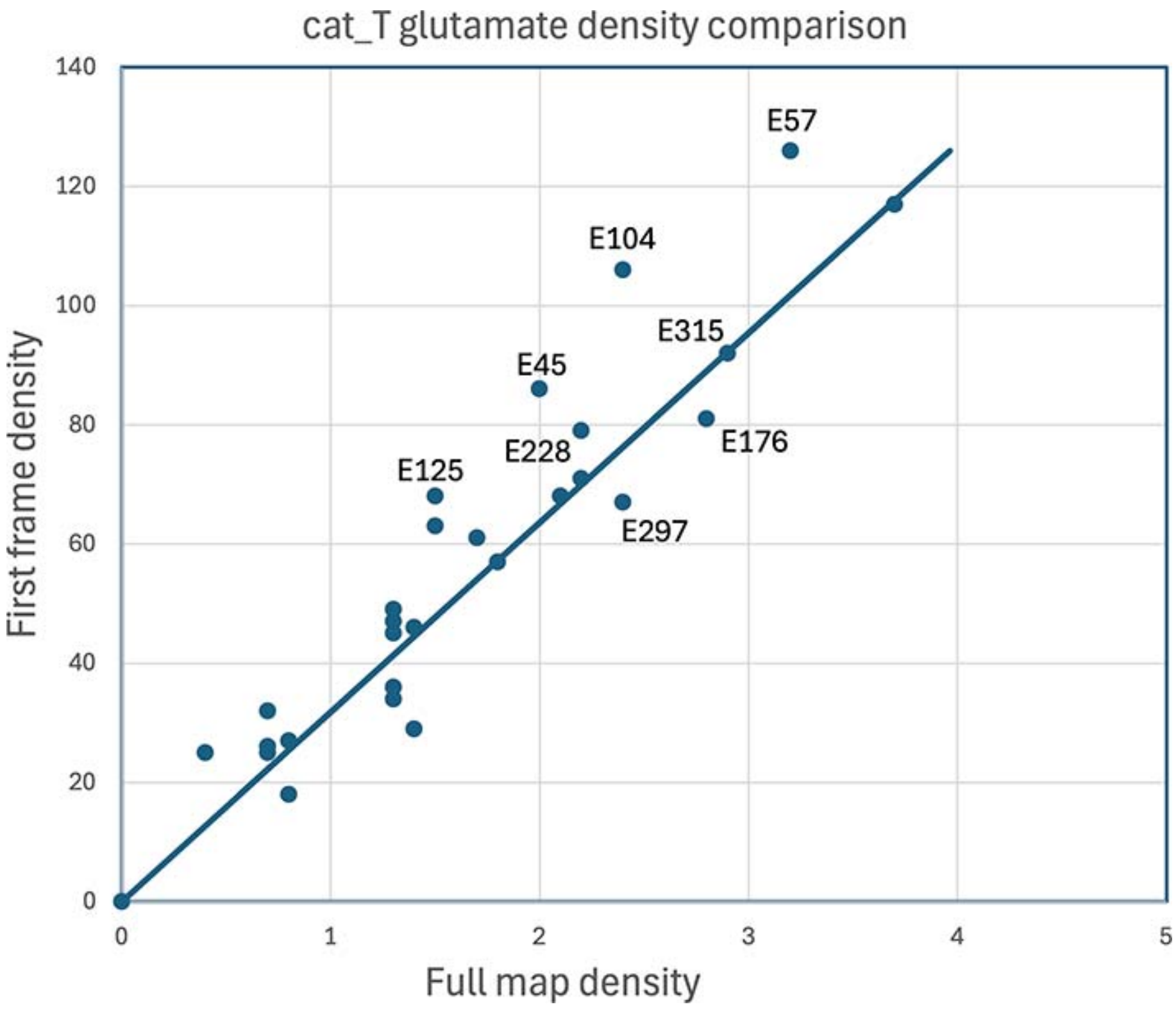

Figure S10 Comparison of cat_T glutamate side-chain densities between a 3D map from only the first frame corresponding to a dose of 1.8 el/Å2 (shown on vertical axis) and the full 3D map produced using the dose-weighted motion-corrected images from RELION (shown on horizontal axis). The labelled residues, which refer to the cat_T maps, are those discussed in the text. The slope of the line takes into account the different map scale factors, at different stages of the RELION processing.
